# Medical College of South Carolina

**Published:** 1883-05

**Authors:** 


### MEDICAL COLLEGE OF SOUTH CAROLINA.

On the evening of March 1st, in Charleston, was held the fifty-fourth annual commencement of the Medical College of South Carolina.

The annual address to the graduates was delivered by

Dr. J. H. Carlisle, President Wofford College, Spartanburg, S. C. Any thing from Dr. Carlisle on such an occasion is always excellent, and we make no apology for the following abstract from the address, as given in the Charleston *News and Courier:*

"You will be expected to remember the debt you owe to your profession. The respect paid to the medical profession is a test of the civilization of a people. A full history of the science of medicine and its leading representative men would touch most of the great currents of our civilization. The intelligent physician is led to study far-reaching questions of many kinds. Every branch of learning has had important contributions from physicians. In Alibine's great Dictionary of English Authors the num ber of medical writers is greater than the number of any one other department. It has been roughly calculated that for the last four hundred years the medical books and pamphlets have averaged two a day. The history of this city will illustrate the activity of the profession. Early in the last century there were more medical and scientific papers and observations published here than in any other American city. While Lining was corresponding with Franklin about strange electrical experiments, Garden and Chalmers were describing in Latin the common weed and flowers around Charleston, for the greatest botanists in the Old World.

"Dr. Garden left the city exactly one hundred years ago, and was welcomed in the scientific circles of London, and given a place among the officers of the Royal Society.

"Later down in the century David Ramsay could write a library of volumes, and yet find time to visit Philadelphia annually to attend the old Congress, and even to preside over it, thus being for a time virtually the President of the Old Confederation. After the adoption of the new Constitution he could go to Columbia and preside over the State Senate, yet all the while keeping up an active correspondence with many distiuguished men of kindred tastes, and keeping fully abreast with his own chosen profession. His celebrated preceptor, Dr. Rush, writing to a friend in this city about Dr. Ramsay, uses these very few but emphatic words, which may well stimulate the aspirations of the young physicians of to-day: 'He talks, writes, and what is more, lives well.' Dr. Ramsay was the first American physician to introduce vaccination, in the case of his son, who died only a few months ago, his long life having measured the years of our century.

"William Charles Wells, author of the 'Theory of Dew,' one of the finest specimens of inductive reasoning in our language, was a native of Charleston, and it may be that some of the beautiful experiments described were tried in his garden in this city.

"Dalcho, the Church historian, spent the first twenty years of this century here as an active physician, before he entered that profession which looks to the moral diseases of our race.

"Sixty years ago Percival, the physician, poet, geologist and genius, spent a few years in Charleston, where he, no doubt, received an impulse from the influences around him.

"These few names are taken from a list which might be enlarged and continued, through the founders of this college down to their successors of to-day. It would show that the intellectual and scientific life of the profession has been fully illustrated here. It should be an educating influence to belong to a historic body which has made contributions to every branch of learning in the past.

"Look, too, at the important questions which now invite the earnest attention of the profession. The multiplied and rapidly-changing diseases to which our stage of civilization is exposed; the growth and influence of great cities; the severe strain of our exacting life on body, mind and heart; the peculiar dangers of certain special trades becoming necessary by our minute division of labor; the wearing labor of some classes, and the scarely less wearing amusements of other classes; the severe competitions of our school life, and the scarcely more trying competitions of maturer life; all these open a wide field of investigation. Add these to the unexampled instance of a strange people before our eyes, rising up to the dignity and responsibility of national life, the oversight of whose physical well-being must be with you for a generation at least. Not only is there earnest work for you and your successors in the number and gravity of the questions before you, but in each science is becoming more minute and exacting. The youngest physician may well feel the humbling yet inspiring impulse of a connection with those who must meet all these questions, while they preserve the national capital of health. This is worth more than the national coal mines, and all the wealth of the wheat zone and the cotton belt.

"Look at your historic, beautiful metropolis. Your three greatest wants of a material kind are, perhaps, a deeper entrance to your harbor, a greater abundance of pure water in the city and exemption from epidemics. Two of these depend on hindrances that can certainly be measured and perhaps can be removed, as they are connected with ascertainable laws of matter. The work of removing these hindrances comes with observation. The results will be known and read of all men, while the connection between cause and effect will be easily traced. With the third want, all is different. To widen your channel so that the crowded immigrant ship can come up to your docks and so that navies may pass safely into your harbor—this may be a gigantic task, but in some respects it is simple. But to close up that gateway so that the pestilence walking in darkness cannot glide in by night, nor the wasting destruction spread over your city at noonday—this is a far different task. How difficult to trace, to measure, to arrest the subtle elements which feed the pestilence! Suppose that for a generation or a century the 'blessed seals that close the pestilence' are not broken. All will agree in recognizing the effect, but they may not agree in assigning the cause. Public meetings, triumphal processions, popular enthusiasm, can do little to help the solitary student who is studying this great problem.

"Young physicians, you see your calling. It has two sides. It has been said that every flower grasps the soil yet seeks the sunshine. And so these mysterious lives of ours must touch both earth and sky. Let your profession have its fullest liberalizing effect on your whole nature and character. Let it build up a completed, generous manhood. Let this completed manhood have its outflow on every side. The physician worthy of his name does more than to set the broken bones and cure the fevers of his neighbors. He teaches, he educates, he enriches. Society feels his presence."

Aside from the intrinsic merit of the address, Dr. Carlisle's delivery was so forcible and eloquent, that his audience hung upon his every word and repeatedly gave exhibition of their appreciation by frequent bursts of applause.

